# Loss of vitamin D receptor induces premature ovarian insufficiency through compromising the 7-dehydrocholesterol-dependent anti-aging effects

**DOI:** 10.3389/fcell.2025.1545167

**Published:** 2025-04-10

**Authors:** Haiyun Chen, Qiuyi Wang, Yi Zhang, Luxi Shangguan, Zhengquan Zhu, Huan Zhang, Xiang Zou, Qinghe Geng, Yanting Wen, Daojuan Wang, Yong Wang

**Affiliations:** ^1^ State Key Laboratory of Analytical Chemistry for Life Science & Jiangsu Key Laboratory of Molecular Medicine, Medical School, Nanjing University, Nanjing, China; ^2^ Liangzhu Laboratory, School of Medicine, Zhejiang University, Hangzhou, China; ^3^ Department of Health Technology and Informatics, Hong Kong Polytechnic University, Kowloon, Hong Kong SAR, China; ^4^ Key Laboratory of Clinical Research of Osteoporosis, Xuzhou Medical University, Xuzhou, China; ^5^ Central Lab, Pizhou Hospital, Xuzhou Medical University, Xuzhou, China; ^6^ Department of Pain, Nanjing Drum Tower Hospital, Affiliated Hospital of Medical School, Nanjing University, Nanjing, China; ^7^ Nanjing University (Suzhou) High-Tech Institute, Suzhou, China

**Keywords:** VDR, premature ovarian insufficiency (POI), granulosa cell, 7-dehydrocholesterol (7-DHC), aging

## Abstract

Vitamin D has the potential to therapeutically affect the endocrine parameters of premature ovarian insufficiency (POI) patients. Previous research has indicated that serum vitamin D levels tend to decline with age and in individuals with POI. However, the precise impact of vitamin D deficiency on female fertility, especially their ovarian function, remains unclear. Vitamin D receptor (VDR) deficiency mice provide a model to investigate the possible effect of vitamin D on female reproduction. In this study, we observed abnormal follicular development in the *Vdr* deficiency mice. This anomaly is associated with reduced expression of anti-Mullerian hormone (AMH) and disrupted aromatase expression that disrupts the hormone secretion. Moreover, our findings indicate that *Vdr* deficiency disturbs redox balance, resulting in oxidative stress in the ovary, which further suppresses granulosa cell function and accelerates ovarian aging. Mechanistically, loss of *Vdr* inhibits *de novo* cholesterol synthesis by transcriptional repression of *Hmgcr*, and the antioxidant and anti-aging effects of the intermediate product 7-dehydrocholesterol (7-DHC) are also decreased. Treatment with 7-DHC effectively reduces ROS levels and alleviates aging in KGN cells deficient in *Vdr*. In conclusion, our results show that *Vdr* deficiency impairs follicle maturation and hormone secretion by accelerating granulosa cell aging, as a result of the reduced antioxidant and anti-aging effect of 7-DHC.

## Introduction

As aging progresses, ovary is one of the earliest impacted organs in female mammals, which is featured by reduced oocyte quality and quantity, and appearance of hormonal dysfunction (Amargant et al., 2020; Yang et al., 2023). In humans, it is reported that female fertility appears to decrease at approximately age 32, which is associated with an increase in oocyte aneuploidies and a diminished capacity for early embryonic development (Nagaoka et al., 2012; Yang et al., 2023). Aging, endocrine disorders, malnutrition and chronic inflammation, all increase the risk of premature ovarian insufficiency (POI) (Panda et al., 2001; Sun et al., 2010; Gou et al., 2023; Touraine et al., 2024).

Vitamin D comes from exposure to sunlight and/or dietary supplements, and it plays a crucial role in adjusting calcium and phosphate metabolism through vitamin D receptor (VDR) while helping to maintain a healthy mineralized skeleton (Panda et al., 2001; Chen et al., 2020). Several studies have confirmed that vitamin D-deficient mice have a shortened lifespan, and develop abnormal organs demonstrating premature aging phenotypes (Artusa et al., 2024). It is generally believed that vitamin D deficiency promotes the senescence of multiple organs, including lung, bone, skin and muscle, and accelerates their functional degeneration (Shuxiang Yu et al., 2021; Zhou et al., 2022). Accumulating evidence supports that vitamin D supplementation is effective in preventing the senescence of various cells (Berridge, 2017; Zhang et al., 2019). The steroid superfamily of receptor, VDR, mediates the biological functions of vitamin D. Once ligand vitamin D binds to VDR, it promotes the formation of heterodimers between VDR and the retinoid X receptor (RXR), which initiates transcription of the target genes.

*Vdr*-deficiency mice have been reported as a mouse model of vitamin D deficiency to demonstrate that female mice with vitamin D deficiency are infertile (Johnson and DeLuca, 2001). VDR has been found in follicular cells; and mRNA levels of VDR and vitamin D metabolic related-aromatase (CYP2R1, CYP27B1 and CYP24A1) were robustly upregulated in breeding seasons compared with non-breeding seasons; and vitamin D content is positive correlation with ovarian steroidogenesis during seasonal changes (Lu et al., 2023). VDR is also reported to relate to POI. VDR mediates the prevention of POI of Gui Shen Wan, a frequently prescribed herbal formula in gynecology, by enhancing vascular regeneration (Zhou et al., 2024). VDR levels are correlated with the activity of luteinized granulosa cells (Yao et al., 2020). Vitamin D promotes follicular development and steroid hormone biosynthesis by upregulating VDR levels (Cheng et al., 2023). Taken together, these findings suggest that VDR is crucial for ovarian development. However, the exact mechanisms by which VDR regulates follicle growth remain unclear.

Current studies have primarily concentrated on how VDR regulates ovarian hormones, while the influence of VDR on ovarian cell metabolism has received minimal attention. Cholesterol is an important precursor substance in the synthesis of androgens, estrogens, progesterone and other sex hormones in theca cells and granulosa cells (de Medeiros et al., 2021; Zhu et al., 2024). Recent studies have demonstrated that disruptions in the mevalonate pathway, which is critical in cholesterol *de novo* synthesis, in aging granulosa cells are linked to defective oocyte meiotic and aneuploidy (Liu et al., 2023a). Granulosa cells also uptake cholesterol from LDL particles *via* LDL receptor-mediated endocytosis (Hu et al., 2010). Cholesterol in theca cells is converted into androgens, which are transported to the granulosa cells and used to synthesize estrogens (Yart et al., 2014). 7-dehydrocholesterol (7-DHC) is an intermediate molecule in the *de novo* synthesis of cholesterol, which can be dehydrogenated and converted into cholesterol by DHCR7 (Li et al., 2024b). A previous study has demonstrated that a decreased concentration of 7-DHC, an intermediate metabolite in cholesterol synthesis, has a significant association with aging (Ghosh et al., 2021). Moreover, it is reported that 7-DHC has a strong antioxidant capacity (Cui and Ye, 2024; Freitas et al., 2024; Li et al., 2024b; Yamada et al., 2024). 7-DHC supplementation can reverse *C. elegans* aging phenotypes (Rottiers et al., 2006). However, whether VDR affects POI through cholesterol synthesis and cholesterol metabolism is unknown. Furthermore, whether 7-DHC mediates the regulation of VDR on POI needs further study.

In this study, vitamin D receptor global knockout mice (*Vdr* ^−/−^) were used to investigate the possible regulatory mechanisms of VDR in the development and functionality of ovary. We discovered that loss of VDR led to abnormal ovarian development and impaired follicle maturation by downregulating antioxidant protein levels and accelerating ovarian cell senescence. Analysis of transcriptome data on the ovaries of mice with a vitamin D-deficient diet showed that cholesterol homeostasis and *de novo* cholesterol synthesis are disrupted. Furthermore, our findings revealed that 7-DHC has the potential to reverse the oxidative aging of granulosa cells as a result of VDR deficiency.

## Result

### *Vdr* deficiency promotes POI and disrupts hormone secretion

**FIGURE 1:**
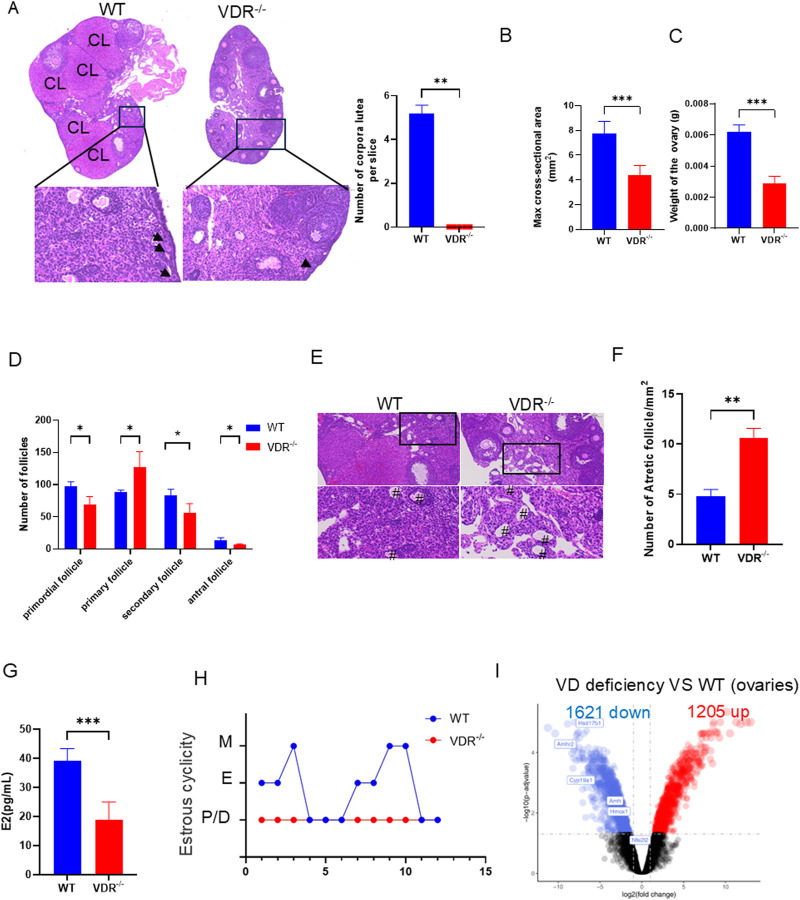
*Vdr* deficiency affected ovarian follicles development and hormone secretion **(A)** Representative microscopic images of the paraffin-embedded ovaries of 8-week-old *Vdr* ^*−/−*^ mice and WT mice stained with hematoxylin-eosin (H&E), and statistical analysis of corpus luteum number with arrows indicating primordial follicles; and CL indicating corpus luteum. **(B)** Max cross-sectional area of ovaries. **(C)** Weight of ovaries in 8-week-old *Vdr* ^*−/−*^ mice and WT mice. **(D)** Statistical analysis of the number of follicles at different developmental stages. **(E)** Representative microscopic images of the paraffin-embedded ovaries of 8-week-old *Vdr* ^*−/−*^ mice and WT mice stained with H&E to reveal atretic follicle. # indicates atretic follicle. **(F)** The number of atretic follicles per mm^2^. **(G)** Serum estrogen levels. **(H)** Estrous cycle of WT and *Vdr* ^*−/−*^ mice. There were three biological replicates in each experiment. Results are expressed as the means ± SEM of six determinations per group. *P < 0.05, **P < 0.01, ***P < 0.001, compared with WT mice using unpaired Student’s t-test. **(I)** Differentially expressed genes (DEG) in WT and vitamin D-deficient ovaries were shown by volcano plot. The upregulated genes (red), and the downregulated genes (blue) with p. adj <0.05.

To explore the impact of VDR on ovarian development, we focused on the comparison between ovaries from the vitamin D receptor (*Vdr*) global knockout mice and those from wild-type (WT) mice. The cross-sectional area of the *Vdr* ^−/−^ ovary at its largest section is remarkably reduced as evidenced by tissue sectioning and hematoxylin and eosin (H&E) staining (Figures 1A, B). The weight of ovaries from *Vdr* ^−/−^ mice was dramatically lower than that of WT mice (Figure 1C). The abnormal follicle development was observed in the H&E-stained ovarian tissue sections as demonstrated by the dramatically reduced number of primordial follicles, absence of the corpus luteum, arrested follicular development in the primary follicles (Figure 1D), and increased numbers of atretic follicles (Figures 1E, F). Serum estrogen levels in *Vdr* deficiency mice also decreased significantly as expected (Figure 1G). Moreover, the estrous cyclicity is disrupted in *Vdr* ^−/−^ mice compared to control mice and mainly arrested in diestrus, which indicates loss of ovarian reproductive function (Figure 1H).

To decipher the underlying mechanism by which vitamin D modulates ovarian development, ovarian transcriptome data (GSE48167) from mice on a vitamin D-deficient diet were analyzed; we identified 2,826 differentially expressed genes (DEGs), including 1,205 upregulated and 1,621 downregulated genes, by comparing mice on the vitamin D-deficient diet and the control diet (Figure 1I). Several downregulated genes could be identified as related to estrogen synthesis, including *Cyp19a1* and *Hsd17b1* (Figure 1I). The decreased mRNA expression levels of *Amh* and *Amhr2*, two ovarian reserve-related genes (Li et al., 2024a), in vitamin D deficient mice indicate a reduction in ovarian reserve (Figure 1I). Together, these findings indicate that VDR is crucial for sustaining ovarian function, and its deficiency may contribute to accelerated POI.

### *Vdr* deficiency causes decreased viability of ovarian granulosa cells

**FIGURE 2:**
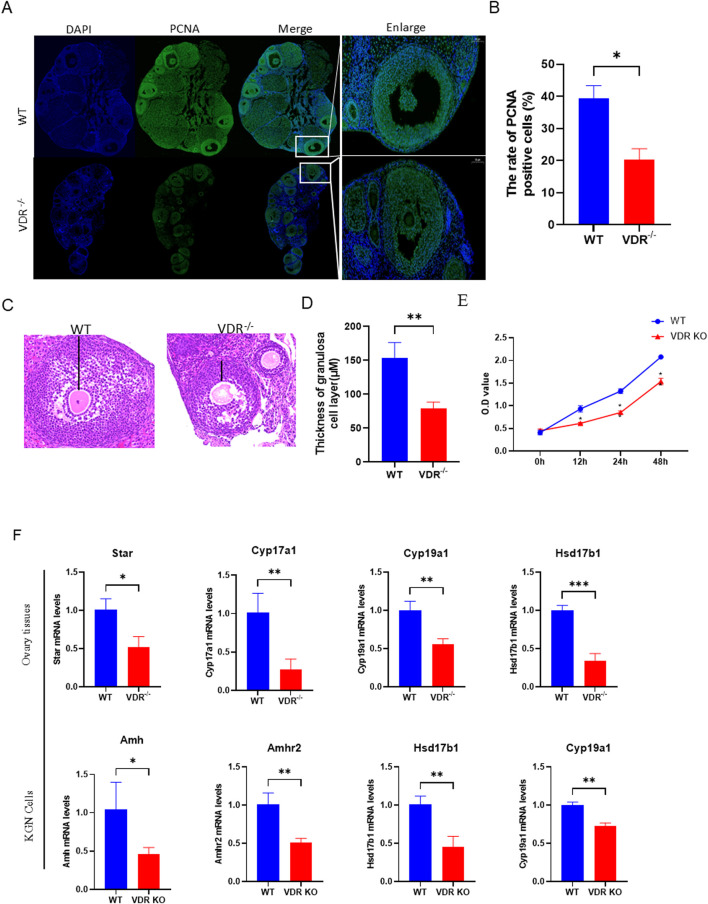
Loss of *Vdr* causes decreased viability of ovarian granulosa cells **(A)** Representative microscopic images of immunofluorescence reveal ovary staining for PCNA, with nuclei stained by DAPI. **(B)** Statistical analysis of the ratio of PCNA-positive cells. **(C)** Representative microscopic images of the paraffin-embedded ovaries of 8-week-old *Vdr* ^*−/−*^ mice and WT mice stained with hematoxylin-eosin (H&E) to reveal the thickness of granulosa cell layer. **(D)** Thickness of granulosa cell layer in ovaries. **(E)** CCK8 assay to determine cell proliferation. **(F)** mRNA levels of *Star, Cyp17a1, Cyp19a1,* and *Hsd17b1* in WT and *Vdr* ^−/−^ ovaries were analyzed using real-time RT-PCR; mRNA levels of *Amh, Amhr2, Hsd17b1,* and *Cyp19a1* in KGN cells were determined using real-time RT-PCR. There were three biological replicates in each experiment. Results are expressed as the means ± SEM of six determinations per group. *P < 0.05, **P < 0.01, ***P < 0.001 compared with WT group using unpaired Student’s t-test.

During the development of the follicle, granulosa cells proliferate and metabolize to provide a rich nutrient microenvironment for oocyte growth. Here, proliferation of granulosa cells was detected by fluorescence staining of proliferating cell nuclear antigen (PCNA) to determine the impact of VDR on granulosa cells. Our results showed that PCNA, a key indicator of cell proliferation (Gao et al., 2024), was significantly downregulated in ovarian granulosa cells in *Vdr* ^−/−^ mice compared with WT mice (Figures 2A, B). We measured the thickness of the granulosa cell layer in WT mice and *Vdr* ^*−/−*^ mice showing similar sizes of oocytes, and the results showed that the thickness of the granulosa cell layer was significantly reduced in *Vdr* ^*−/−*^ mice (Figures 2C, D). Expression of genes related to steroid hormone synthesis were detected in *Vdr* ^−/−^ ovaries using RT-PCR. *Star* and *Cyp17a1*, which are specifically expressed in theca cells and catalyze the synthesis of androgens from cholesterol, were significantly downregulated in *Vdr* ^−/−^ ovary tissues (Figure 2F). *Cyp19a1* and *Hsd17b1*, which are specifically expressed in ovarian granulosa cells and catalyze the synthesis of estrogen from androgens derived from theca cells, were also significantly downregulated in *Vdr* ^−/−^ ovary tissues (Figure 2F). These results suggest that decreased viability of ovarian granulosa cells is observed in VDR-deficient ovaries. To further confirm this conclusion, the CRISPR-Cas9 system was used to knockout *Vdr* in KGN cells to confirm the effect of VDR on the activity of granulosa cells. We found that the cell proliferative capacity was significantly reduced in *Vdr* knockout KGN cells (VDR KO) compared with WT KGN cells using CCK-8 (Figure 2E). Moreover, expression of *Amh*, *Amhr2, Cyp19a1* and *Hsd17b1* were significantly downregulated in VDR KO KGN cells (Figure 2F). These findings demonstrated that VDR deficiency inhibits proliferation of granulosa cells, downregulates estrogen synthesis and diminishes ovarian reserve both *in vivo* and *in vitro*.

### *Vdr* deficiency disrupts redox homeostasis in ovarian granulosa cells

**FIGURE 3:**
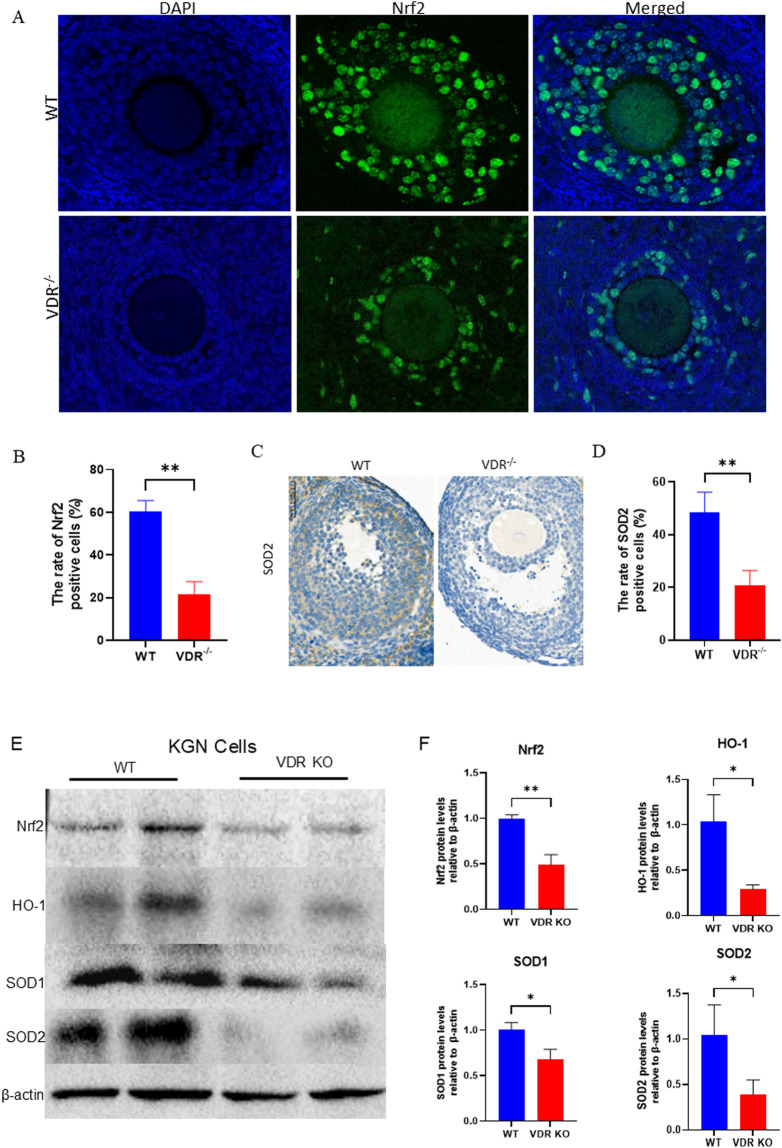
Redox homeostasis was disrupted in the *Vdr*-deficient granulosa cells **(A)** Representative microscopic images of immunofluorescence reveals ovary staining for Nrf2, with nuclei stained by DAPI. **(B)** Statistical analysis of the ratio of Nrf2-positive cells. **(C)** Representative micrographs of immunohistochemical staining show ovary staining for SOD2. **(D)** Percentage of positive cells for SOD2. **(E)** KGN cell extracts were analyzed using western blots showing Nrf2, HO-1, SOD1, and SOD2 protein levels. β-actin was considered as the loading control. **(F)** Densitometric analysis was used to assess protein expression levels relative to β-actin. There were three biological replicates in each experiment. Results are expressed as the means ± SEM of six determinations per group. *P < 0.05, **P < 0.01, ****P < 0.0001, compared with the WT group using unpaired Student’s t-test.

Our previous study demonstrated that vitamin D deficiency could cause oxidative stress and premature tissue aging (Zhou et al., 2022). Ovaries from *Vdr* ^−/−^ and WT mice were examined for their production of antioxidant proteins to investigate whether VDR deficiency disrupts redox homeostasis in ovaries. Nuclear factor erythroid 2-related factor 2 (Nrf2), a key transcription factor that activates antioxidant enzymes (Bae et al., 2024), such as heme oxygenase-1 (HO-1) and superoxide dismutase (SOD), to maintain redox homeostasis, was significantly downregulated in ovarian granulosa cells in *Vdr* ^−/−^ mice compared with WT mice confirmed by fluorescence staining of Nrf2 (Figures 3A, B). SOD2 was downregulated in granulosa cells of antral follicles in *Vdr* ^−/−^ mice compared with WT mice as demonstrated by immunohistochemical staining (Figures 3C, D). To further confirm these results, we measured the antioxidant protein, including Nrf2, HO-1, SOD1 and SOD2, levels in VDR KO KGN cells. A marked decrease in Nrf2 protein levels in the VDR KO KGN cells was observed; SOD1/2 and HO-1 protein levels were also decreased significantly as expected confirmed by Western blot (Figures 3E, F). Overall, our findings suggest that VDR deficiency diminishes the antioxidant capacity in ovarian granulosa cells by suppressing the Nrf2 signaling pathway.

### *Vdr* deficiency promotes aging of ovarian granulosa cells

**FIGURE 4:**
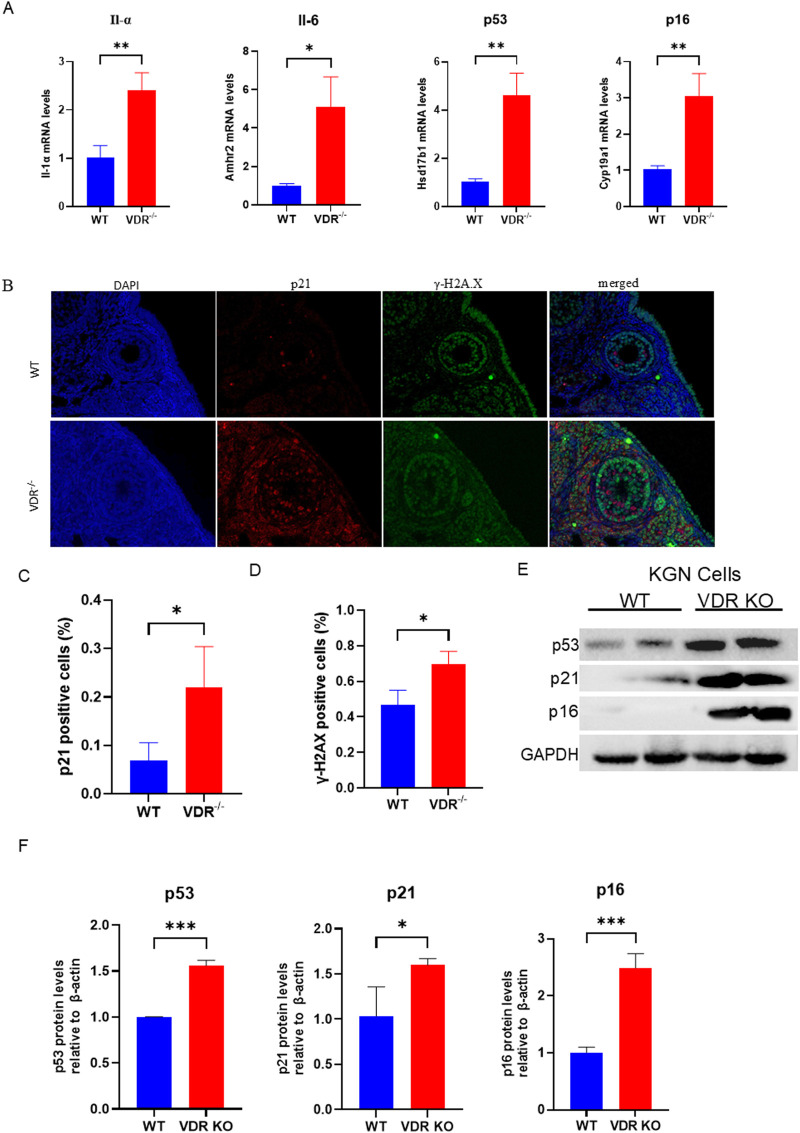
Loss of *Vdr* promotes granulosa cell aging **(A)** mRNA levels of *Il-α, Il-6, p53,* and *p16* in WT and *Vdr* ^−/−^ ovaries were analyzed using real-time RT-PCR, calculated as relative to β-actin mRNA. **(B)** Representative microscopic images of immunofluorescence indicate ovary staining for p21 and γ-H2A.X. **(C)** Statistical analysis of the ratio of p21-positive cells. **(D)** Statistical analysis of the γ-H2A.X positive cell ratio. **(E)** KGN cell extracts were analyzed using western blots to reveal p53, p21, and p16 protein levels. GAPDH was considered as the loading control. **(F)** Densitometric analysis was used to assess protein expression relative to GAPDH. There were three biological replicates in each experiment. Results are expressed as the means ± SEM of six determinations per group. *P < 0.05, **P < 0.01, ***P < 0.001, compared with the WT group using unpaired Student’s t-test.

Oxidative damage usually induces DNA damage and then leads to cell senescence. Therefore, after confirming that absence of VDR causes downregulation of antioxidant enzymes, we investigated whether VDR deficiency would lead to cell senescence. Our results showed that senescence-associated genes, including the pro-inflammatory factors Il1a, Il6, and the aging-related markers p53 and p16 (Wu et al., 2024), were significantly upregulated in the ovaries of *Vdr* ^−/−^ mice confirmed by RT-PCR (Figure 4A). Moreover, we analyzed the levels of aging and the DNA damage-related molecules in *Vdr* ^−/−^ ovarian tissues using immunofluorescence staining of p21 and γ-H2A.X. The results revealed a significant increase in the p21-and γ-H2A.X-positive cell levels in the ovarian granulosa cells of *Vdr* ^−/−^ mice (Figures 4B–D). Furthermore, the protein expression levels of p53, p21 and p16 were increased significantly in VDR KO KGN cells compared with WT KGN cells (Figures 4E, F). These results demonstrated that VDR deficiency causes ovary aging, in particular the aging of granulosa cells.

### Vitamin D deficiency disrupts cholesterol *de novo* synthesis

**FIGURE 5:**
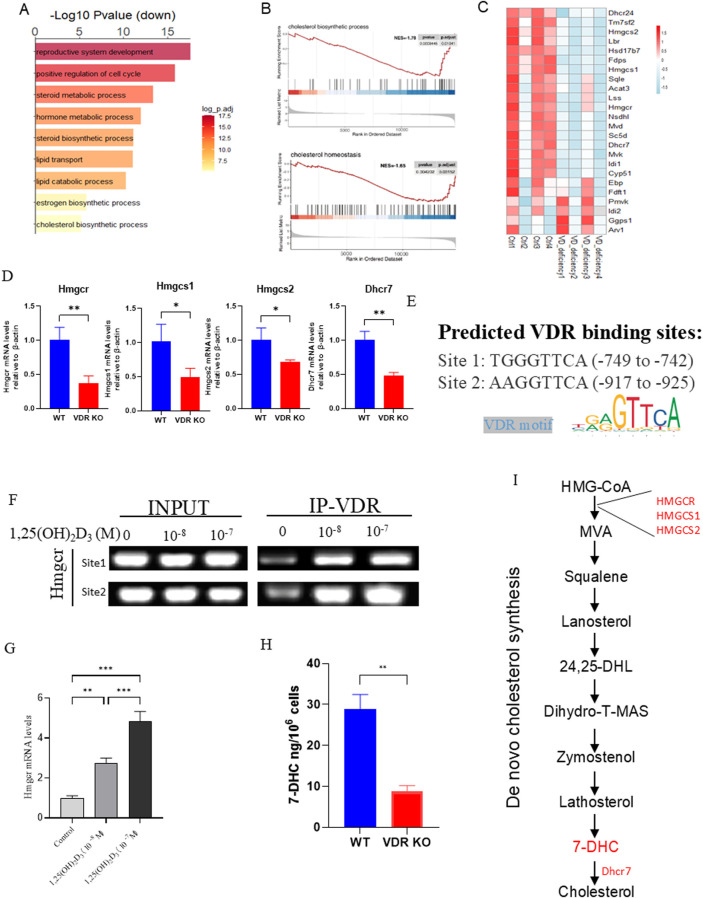
Vitamin D deficiency affected cholesterol *de novo* synthesis **(A)** Gene Ontology (GO) analysis of vitamin D-deficient and WT ovaries. **(B)** Genes related to cholesterol biosynthetic processes and cholesterol homeostasis were identified by gene set enrichment analysis (GSEA). **(C)** Heatmap plot showes the expression of cholesterol *de novo* synthesis-related genes in vitamin D-deficient and WT ovaries. **(D)** mRNA levels of *Hmgcr, Hmgcs1, Hmgcs2,* and *Dhcr7* in VDR KO KGN cells were analyzed using real-time RT-PCR, calculated as relative to β-actin mRNA. **(E)** VDR binding sites in the *Hmgcr* promoter region was predicted by JASPAR. **(F)** Gel-image for ChIP PCR. **(G)** *Hmgcr* mRNA levels in KGN cells following treatment with different concentrations of 1,25(OH)_2_D_3_ were analyzed using real-time RT-PCR, calculated as relative to β-actin mRNA. **(H)** 7-DHC levels in KGN cells were measured using ELISA. **(I)** Mechanism diagram of *de novo* cholesterol synthesis. There were three biological replicates in each experiment. Results are expressed as the means ± SEM of six determinations per group. *P < 0.05, **P < 0.01, ***P < 0.001, compared with the control group using unpaired Student’s t-test.

To investigate the underlying mechanism by which VDR deficiency leads to POI, we conducted bioinformatics analysis on publicly available datasets comparing mice with a vitamin D-deficient diet and a normal diet (GSE48167). Gene Ontology (GO) analysis revealed that, among the downregulated DEGs, “reproductive system development,” “estrogen biosynthetic process,” “steroid metabolic process” and “cholesterol biosynthetic process” were identified as the top GO pathways in vitamin D deficiency ovary (Figure 5A). Furthermore, GSEA analysis revealed that “cholesterol biosynthetic process”- and “cholesterol homeostasis”-related genes were downregulated in vitamin D deficiency ovary compared with controls (Figure 5B). Taken together, these data suggest that cholesterol metabolism is disordered in vitamin D deficiency ovaries. We further analyzed the expression of genes related to *de novo* cholesterol synthesis, and the result showed that these genes are downregulated in vitamin D deficiency ovaries (Figure 5C). RT-PCR was carried out to verify the significantly downregulated expression of cholesterol *de novo* synthesis-related enzymes, including *Hmgcr*, *Hmgcs1*, *Hmgcs2* and *Dhcr7* in VDR KO KGN cells (Figures 5D, I). *Hmgcr* is the rate-limiting enzyme in *de novo* cholesterol synthesis, and there are two VDRE-like sequences at 2 kb upstream of the *Hmgcr* gene identified by bioinformatics analysis, which implies that VDR probably regulates *Hmgcr* transcription (Figures 5E, I). To determine whether vitamin D regulates the *Hmgcr* via the VDR at a transcriptional level, KGN cells treated with or without 1,25(OH)_2_D_3_ (active form of vitamin D) were used to execute chromatin immunoprecipitation (ChIP). The result of the ChIP-PCR assay demonstrated that VDR could bind to the promoter of *Hmgcr* gene; and 1,25(OH)_2_D_3_ could enhance the combination in a dose-dependent manner (Figure 5F). Moreover, 1,25(OH)_2_D_3_ treatment significantly upregulated *Hmgcr* mRNA expression in KGN cells in a dose-dependent manner (Figure 5G). These results suggest that vitamin D transcriptionally regulates cholesterol synthesis via VDR. 7-DHC is an intermediate metabolite of cholesterol synthesis (Figure 5I) and has been reported to have strong antioxidant capacity (Li et al., 2024b), and 7-DHC was significantly downregulated in VDR KO KGN cells (Figure 5H). In conclusion, vitamin D deficiency causes abnormal levels of cholesterol and its derivatives via VDR mediated transcriptional regulation of the expression of HMGCR.

### Supplementation of 7-DHC alleviates aging and promotes anti-oxidation

**FIGURE 6:**
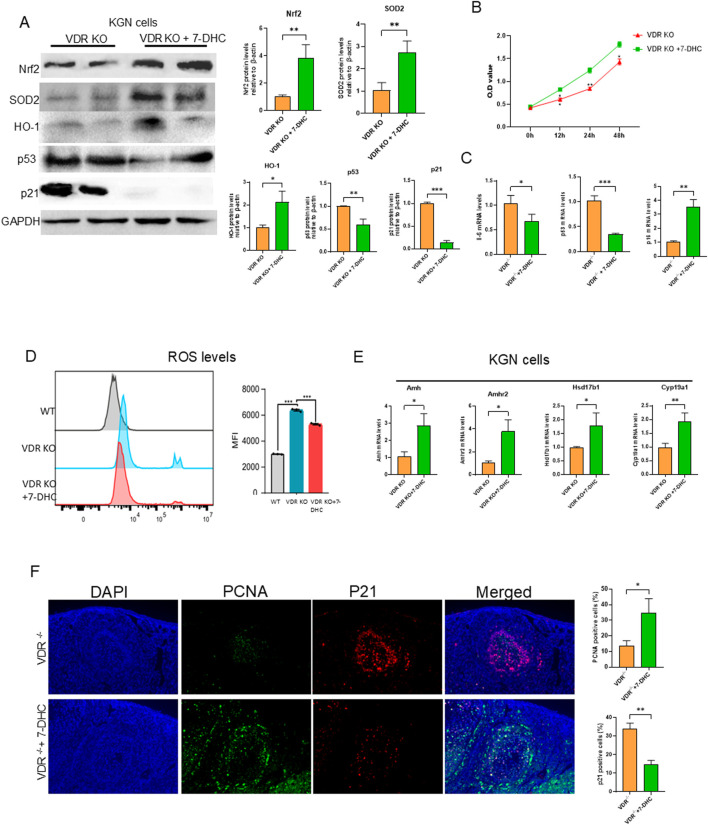
7-DHC supplementation delays oxidative aging of granulosa cells induced by *Vdr* deficiency Mice and KGN cells were treated with 7-DHC. **(A)** KGN cell extracts were conducted western blots showing Nrf2, SOD2, HO-1, p53, and p21 protein levels. GAPDH was considered as the loading control. Densitometric analysis was used to assess protein expression relative to GAPDH. **(B)** CCK8 experiment determines the effect of 7-DHC on cell proliferation. **(C)** Il-6, p53, and p21 mRNA levels in ovary tissue by real-time RT-PCR, calculated as relative to β-actin mRNA. **(D)** The ROS level was measured using flow cytometry and mean fluorescence intensity (MFI) showed positive areas for ROS. **(E)** mRNA levels of *Amh, Amhr2, Hsd17b1,* and *Cyp19a1* in VDR KO KGN cells were analyzed using real-time RT-PCR, calculated as relative to β-actin mRNA. **(F)** Representative microscopic images of immunofluorescence showed ovary staining for PCNA and p21. Statistical analysis of PCNA- and p21-positive cell ratio. There were three biological replicates in each experiment. Results are expressed as the means ± SEM of six determinations per group. *P < 0.05, **P < 0.01, ***P < 0.001. Compared with VDR KO KGN cells or *Vdr* ^*−/−*^, unpaired Student’s t-test.

7-DHC, an intermediate product of *de novo* synthesis of cholesterol, has antioxidant functions (Freitas et al., 2024; Li et al., 2024b; Yamada et al., 2024), and has been reported to be associated with aging (Rottiers et al., 2006; Ghosh et al., 2021). To verify whether 7-DHC could alleviate ovarian granulosa cell aging caused by VDR deficiency, we treated VDR KO KGN cells with 7-DHC and measured protein levels of antioxidant- and aging-related indicators. Our data confirmed that supplementing VDR KO KGN cells with 7-DHC significantly increased the protein levels of Nfr2, SOD2 and HO-1, and decreased the protein levels of p53 and p21 compared with VDR KO KGN cells without supplementation (Figure 6A). Moreover, 7-DHC could promote VDR KO KGN cell activity confirmed by CCK8 (Figure 6B). Furthermore, VDR KO KGN cells had increased reactive oxygen species (ROS) levels compared with WT KGN cells, and 7-DHC treatment could decrease the ROS levels in VDR KO KGN cells as determined by flow cytometric analysis (Figure 6D). We were intrigued whether 7-DHC could restore the ability of granulosa cells to express the genes that promote follicle development and inhibit the recruitment of primordial follicles in VDR KO granulosa cells; our data showed that mRNA expression of *Amh, Amhr2, Hsd17b1* and *Cyp19a1* was significantly increased in 7-DHC-treated VDR KO KGN cells (Figure 6E). To determine whether 7-DHC could delay aging and increase granulosa cell viability *in vivo*, *Vdr* ^*−/−*^ mice were treated with or without 7-DHC for 4 weeks. Then, ovaries were used to analyze the aging phenotype. Our data demonstrated that PCNA-positive cells increased in 7-DHC treatment ovaries, and p21-positive cells decreased in 7-DHC treatment ovaries (Figure 6F); mRNA levels of IL-6, p53 and p16 decreased in 7-DHC treatment ovaries (Figure 6C). Based on these results, we concluded that 7-DHC could increase the antioxidant protein levels and delay aging induced by VDR deficiency, and 7-DHC treatment could promote ovarian estrogen secretion and strengthen ovarian reserve both *in vivo* and *in vitro*.

## Discussion

Premature ovarian insufficiency (POI), commonly referred to as premature ovarian failure, is a disorder of ovary in humans marked by the exhaustion of ovarian follicles before the age of 40 (Chen et al., 2023; Liu et al., 2023b). Nevertheless, the mechanisms underlying POI are still largely unclear. A clinical study revealed that women with POI frequently have low levels of vitamin D, which are inversely correlated with follicle-stimulating hormone (FSH) levels (Kebapcilar et al., 2013). It is reported that appropriate vitamin D supplementation may improve POI by inhibiting neutrophil extracellular traps (Chen et al., 2023). *Vdr* deficiency mice showed infertility phenotype, which could be partially reversed by high dietary calcium treatment (Johnson and DeLuca, 2001). These imply that Vitamin D/VDR plays an important role in ovarian function, but the underlying mechanisms remain unclear. In this study, *Vdr* global knockout mice were employed as a vitamin D deficiency mouse model. We demonstrate that knocking out the *Vdr* both in primary granulosa cells and KGN cells significantly inhibited cell proliferation, promoted cell aging, and increased oxidative stress, leading to a POI-like phenotype and infertility in female *Vdr* knockout mice. Mechanistic studies have shown that supplementation with 7-DHC, which is downregulated by vitamin D deficiency, prevents ovarian granulosa cell aging through anti-oxidation.

The bioinformatics analysis of the ovaries transcriptome data (GSE48167) from mice on a vitamin D-deficient diet showed that vitamin D insufficiency downregulated mRNA levels of *Amh* [an ovary-produced hormone that is a marker of ovarian reserve (Nyström et al., 2024; Touraine et al., 2024)] and *Amhr2* (Anti-Mullerian Hormone Receptor Type 2), which indicates premature ovarian insufficiency in vitamin D-deficient mice. Granulosa cell proliferative capacity was decreased as shown by fluorescent staining of PCNA on ovaries and CCK-8 analysis of KGN cells, which supports previous findings that both low and high doses of vitamin D3 stimulate granulosa cell proliferation in hen follicles (Wojtusik and Johnson, 2012). Nrf2 is essential for activating antioxidant enzymes in cells for maintaining cellular redox homeostasis (Liu et al., 2024). Our results demonstrated that *Vdr* deficiency downregulated mRNA levels of Nrf2 and its downstream target, HO-1. Protein expression levels of Nrf2, HO-1, SOD1 and SOD2 were downregulated in both *Vdr* ^*−/−*^ ovaries and *Vdr* KO KGN cells. Increased protein levels of p21 and γ-H2A.X in ovarian cells of *Vdr* ^−/−^ mice, and increased levels of p16, p21 and p53 in VDR KO KGN cells indicated that ovarian cells developed aging upon deletion of *Vdr*. These results showed that *Vdr* knockout increased levels of oxidative stress and accelerated cell senescence in ovarian granulosa cells and KGN cells.

To further study the mechanism of POI resulting from *Vdr* deficiency, ovarian transcriptome data from mice on a vitamin D-deficient diet (GSE48167) were analyzed. The GO and GSEA analysis showed that *de novo* cholesterol synthesis is inhibited and cholesterol homeostasis is disrupted in ovaries following vitamin D deficiency. Previous reports showed that intermediate metabolite products from cholesterol biosynthesis and amino acids in GCs were essential for oocyte growth and maturation (Su et al., 2009). Disruption of cholesterol synthesis in ovaries is detrimental to female reproduction (Varik et al., 2024). The abnormal mevalonate pathway, a crucial pathway in cholesterol metabolism, in granulosa cells causes aging-related meiotic defects in oocytes (Liu et al., 2023a). These findings imply that cholesterol and intermediate metabolites from cholesterol biosynthesis in granulosa cells may influence ovarian development and function. Several types of experiments have proven that 7-DHC, intermediates in cholesterol synthesis, have a strong antioxidant effect and could inhibit lipid peroxidation in cells, which protects cells from ferroptosis (Sun et al., 2023; Freitas et al., 2024; Yamada et al., 2024). DHCR7 facilitates the conversion of 7-DHC into cholesterol, and its inhibition leads to reduced cholesterol levels and elevated 7-DHC levels (Shin et al., 2019). However, several reports have shown that inhibiting DHCR7 raises the levels of 7-DHC, which is significantly more prone to oxidation than cholesterol (Speen et al., 2019). Knockdown of DHCR7 impaired cell proliferation, triggered apoptosis, and disrupted mitochondrial function (Wang et al., 2024). Moreover, increased expression of DHCR7 leads to cholesterol accumulation and enhances the expression of CYP19A1, thereby promoting the production of steroid hormones (Li et al., 2023). Stewart et al. reported that progesterone secretion is reduced when GCs are treated with an inhibitor capable of conversting 7-DHC to cholesterol (Stewart et al., 1982). These controversies highlight the controversial role of 7-DHC in ovarian cells. Here, our results showed that treatment of *Vdr* ^−/−^ KGN cells with 7-DHC reduced aging-related protein expression of p53 and p21, reduced intracellular ROS levels and increased antioxidant enzymes Nrf2, SOD2 and HO-1 protein levels, which suggests that 7-DHC could protect granulosa cells from oxidative stress and aging. In addition, 7-DHC treatment promoted the proliferation of VDR KO KGN cells and increased ovarian reserve-related mRNA expression of *Amh* and *Amhr2*; and estrogen synthesis-related mRNA levels of *Hsd17b1* and *Cyp19a1*. Furthermore, a study has shown that weaned pups of *Vdr* deficiency mice to a high calcium-containing diet could restore their fertility, whether calcium metabolism affects granulosa cell activity and the effect of calcium on ovarian development deserves further study (Johnson and DeLuca, 2001). In conclusion, our data have demonstrated that 7-DHC plays a protective role in preventing ovarian granulosa cell senescence and promoting estrogen secretion.

## Conclusion

*Vdr* deficiency female mice develop abnormal ovaries; granulosa cells showed an aging phenotype of decreased proliferation activity and reduced estrogen secretion capacity. The underlying mechanism is cholesterol metabolism disorders in *Vdr* deficiency mice.

Treatment of VDR-deficient granulosa cells with 7-DHC could delay VDR deficiency granulosa cell senescence through upregulated Nrf2 to resist oxidative stress. Thus, VDR plays a key role in regulating granulosa cell aging and ovarian aging.

## Experimental procedures

### Mice and genotyping

Vdr+/− mice with a C57BL/6J background were donated by Professor Jin Jianliang, Vdr−/− mice were generated through the breeding of heterozygous mice (Vdr+/−), and the detailed identification methods are described in Supplementary Material 1. The mice were treated by gavage with 5 mg/kg 7-DHC. The mice used in this experiment were housed under appropriate temperature and humidity conditions. Isoflurane was used to euthanize mice, all mouse procedures were conducted under humane process, and the procedure followed the guidelines of the Animal Ethics Committee of Nanjing University (Permit Number: IACUC-2103061).

### Monitoring of estrous cycles

Vaginal smears from female mice were collected for H&E staining at 5 p.m. every day for 12 days. Proestrus, estrus, and diestrus were evaluated based on the epithelial cell morphology and the number of leukocytes in the smear according to the previous description (Byers et al., 2012).

### Serum estrogen test

Concentrations of estrogen in serum from mice were detected using ELISA kits (Yifeixue Biotechnology) following the manufacturers’ instructions.

### Cell culture and treatment

KGN cells, human ovarian granulosa cells, is a steroidogenic ovarian granulosa tumor cell line. The cells retain most of the physiological activities of ovarian granulosa cells, including the expression of functional follicle-stimulating hormone (FSH) receptors and steroidogenesis similar to that observed in normal granulosa cells. KGN was cultured in DMEM/F12 medium supplemented with 10% FBS and 1% penicillin-streptomycin solution, maintained at 37°C in a 5% CO2/air environment. KGN cells were cultured in 6-well plates. When cells grow to 80% density, 7-DHC were added (25 μM), they were incubated for 24 h, followed by the harvesting and extraction of total proteins or total RNA.

### Data acquisition and data processing

The ovaries’ transcriptome data from mice on a vitamin D-deficient diet are from Gene Expression Omnibus under the accession number GSE48167. Differentially expressed genes (DEGs) with |log2foldchange|≥1 and p. adj<0.05 were determined by the limma package using the R program. Gene Ontology (GO) and Gene set enrichment analysis (GSEA) were analyzed using the “ClusterProfiler” R package. The “ggplot2” R package was applied for plotting the bar chart.

### Vdr knockout KGN cell generation

CRISPR-Cas9 system was conducted to generate Vdr knockout cells as previously described (Xie et al., 2017). Two sgRNA targeting VDR gene were subcloned into the epiCRISPR vector, separately. Then, the two plasmids were co-transfected into KGN cells. After 48 h of transfection, the cells were selected using puromycin (0.5 μg/mL) for 5 days, then single cells were selected and planted into 96-well plates. Vdr knockout cells were validated by Western blot analysis. The sequences of the sgRNA are listed here. sgVDR1 F 5′-caaagtctccagggtcaggc-3′ R 5′-gcctgaccctggagactttg-3′; sgVDR2 F 5′-tcacaggtcatagcattgaa-3′ R 5′-ttcaatgctatgacctgtga-3′.

### RNA extraction and real-time RT-PCR

The method of extraction and reverse transcription of total RNA followed the instructions provided by the reagent manufacturer (#15596, Invitrogen Inc.), and quantification of target mRNA by RT-PCR was performed as described in Supplementary Material 1. Information of primers is shown in Supplementary Table 1.

### Western blots

Detailed steps of western blots are described in Supplementary Material 1. The primary antibodies used are listed here. Nrf2 (#12721, Cell Signaling Technology, United States), p16 (ab211542, Abcam, United States), p53 (sc-126, Santa Cruz Biotechnology Inc., United States), p21 (#2946, Cell Signaling Technology, United States), HO-1 (#70081, Cell Signaling Technology, United States), SOD1 (#2770, Cell Signaling Technology, United States), and SOD2 (#13141, Cell Signaling Technology, United States). β-actin or Gapdh was the loading control for the cytoplasmic fraction and total cell protein.

### Immunofluorescent staining or immunohistochemical staining

Primary antibodies against PCNA (10205-2-AP, Proteintech Biotechnology Inc., China), Nrf2 (#12721, Cell Signaling Technology, United States), p21 (#2946, Cell Signaling Technology, United States), γ-H2A.X (AF1201, Beyotime Biotechnology, Shanghai, China) were used. Fluorescent secondary antibodies were used (Dylight488 or Dylight594 conjugated secondary antibody); please refer to the Supplementary Material 1 for detailed methods.

### Intracellular ROS analysis

5 μM CM-H2DCFDA (Beyotime) was added to the 6-well plate containing KGN cells and incubated in a 37°C cell culture incubator for 30 min, then the cells were washed three times with serum-free cell culture medium to fully remove the CM-H2DCFDA that did not enter the cells. Flow cytometry (CytoFLEX) was used to detect intracellular ROS levels.

### CCK8 assay

The CCK8 method was used to detect cell proliferation according to the instructions provided by the reagent manufacturer (#C0038, Beyotime Institute of Biotechnology, Shanghai, China), please refer to the Supplementary Material 1 for detailed methods.

### Chromatin immunoprecipitation assay

The ChIP experiment was performed using a kit, The ChIP experiment was performed using a kit (#17295, Sigma-Aldrich United States). For specific methods, refer to the instructions of the reagent manufacturer. Antibodies against Vdr (Abcam, ab3508), and rabbit IgG (#PP64; Millipore) were used to incubate chromatin samples. Primers used are listed here. Site1-F:ACAGGTTCTAGGCTCTCGGT, Site1-R: CTGTGGTTGGCATGGGAGAT; Site2-F: GGGAACAGTAGAACCAGCCT, Site2-R: AGGACAGGAATTGTATTTCACTCAA.

### Statistical analysis

GraphPad Prism software (Version 9.4; GraphPad Software Inc., San Diego, CA, United States) was used for statistics, the data normal distributions and homogeneity test of variances were determined by Shapiro-Wilk test and Levene’s test, respectively, all measurement data are presented as mean ± SEM. Two-tailed Student’s t-test was used for statistical test between two groups, one-way ANOVA was used to compare the means of three or more groups followed by Tukey’s *post hoc* test. P-value less than 0.05 was considered statistically significant.

## Data Availability

Publicly available datasets were analyzed in this study. This data can be found here: Gene Expression Omnibus under the accession number GSE48167.
