# Supplementary material for: Loss of vitamin D receptor induces premature ovarian insufficiency through compromising the 7-dehydrocholesterol-dependent anti-aging effects
Source: Front Cell Dev Biol. 2025 Apr 10;13:1545167. doi: 10.3389/fcell.2025.1545167 (PMC12018433; doi:10.3389/fcell.2025.1545167)
Supplement: Supplementary file 1 [file DataSheet1.pdf]

RNA isolation and real-time RT–PCR

Total RNA was extracted from cultured KGN or ovaries using TRIzol reagent (Vazyme) according to the manufacturer's instructions. Complementary DNA (cDNA) was synthesized using Synthesis SuperMix (Vazyme). The real-time RT-PCR was carried out by a Quantstudio 12 K Flex (Applied Biosystems) using the SYBR qPCR Master Mix (Vazyme). Gapdh was amplified at the same time to normalize gene expression. Groups at least six mice were examined, and each experiment was repeated three times to determine relative gene expression differences. The PCR primer sequences used in this study are shown in the Table S1.

Mouse genotype identification

Genomic DNA was extracted from the newborn 7-day-old mice tails using a DNA extraction kit (Qiagen) and subjected to PCR to confirm the homozygous knockout status. Primers used to identify the genotype are listed here. VDR-WT: CTCCATCCCCATGTGTCTTT; VDR-mutant: CACGAGACTAGTGAGACGTG; VDR-common: TTCTTCAGTGGCCAGCTCTT.

SI Fig 1

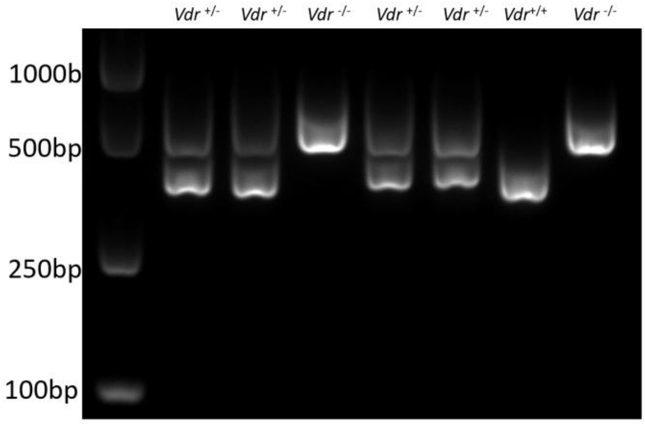

Western blots

KGN cell proteins were extracted using protein lysis buffer (FUDE BIOLOGICAL TECH, HangZhou, China). Next, equal amounts of protein were loaded per sample and separated by 10% SDS-PAGE electrophoresis before being transferred to a PVDF membrane. The membrane was blocked with 5% skim milk in TBST with Tween-20 for 1 h and then incubated with primary antibodies at 4°C overnight. After several washes, the membrane was incubated with corresponding secondary antibodies for 1 h at room temperature. Finally, protein bands were visualized using an enhanced chemiluminescence detection system (Bio-Rad), and protein expression levels were calculated by normalizing to Gapdh/β-actin levels using Image J software.

Immunofluorescence/ Immunohistochemical staining

Using xylene, the ovary paraffin sections were deparaffinized and subsequently rehydrated. For immunocytochemical staining, after multiple washes with PBS, the ovary paraffin sections were blocked using 5% BSA for 1 h at room temperature. The sections were then incubated overnight at 4°C with the PCNA(1;200), p21(1:200), γ-H2A.X(1:100)antibody, followed by the corresponding secondary antibody to detect immunoreactivity.

For immunohistochemistry staining, dewaxed and rehydrated paraffin-embedded sections were incubated with 6% hydrogen peroxide to block endogenous peroxidase activity and then washed in TBST. The slides were then incubated at 4°C overnight with the primary antibodies to SOD2. After rinsing with TBST, tissues were incubated with secondary antibodies (biotinylated goat anti-rabbit IgG and goat anti-mouse IgG; Sigma). Sections were then washed and incubated with the Vectastain Elite ABC reagent (Vector Laboratories) for 30 min. Staining was done using 3,3-

diaminoben-zidine (2.5 mg/ml) followed by counterstaining with Mayer's hematoxylin.

CCK8 assay

Cell proliferation was analyzed using cell counting kit-8 (CCK-8) assay kits (Beyotime). KGN cells were seeded in 96-well plates at 2000 cells per well. KGN cells were incubated for 0, 12, 24 or 48 hours. CCK-8 (10 µl) was added to each well and cultures were incubated at 37 °C for 1 hour. Cells were detected by spectrophotometry at 450 nm absorbance following the manufacturer’s instructions.

Follicle counting and GC characterization

Ovarian samples were fixed in 4% paraformaldehyde overnight, embedded in paraffin, and cut into 5-µm-thick sections. Tissue sections were stained with hematoxylin-eosin (H&E) to count the number of follicles. The follicle stage was classified by its morphological characteristics according to a previous study[1]. Briefly, A primordial follicle contained an oocyte with a diameter of less than 20µm; A primary follicle contained a larger oocyte and one-layer cubical ovarian granulosa cells; A secondary follicles had multilayer GCs; Antral follicles had antral cavities. The number of every follicle stage in 5-µm ovarian sections was counted in every fifth section and multiplied by 5 to calculate all follicles in each ovary. Only follicles containing visible oocyte nuclei in each section were counted to avoid repetitive counting. The thickness of the GCs was acquired by averaging the maximum and minimum thickness of layers using Olyvia software (Olympus).

Quantification of granulosa cell thickness

The quantification of granulosa cell thickness was performed following the reported methods [23, 60]. Briefly, the sections were stained with hematoxylin, follicles with clear oocyte nuclei were

measured the granulosa cell thickness with Olyvia (Olympus) software.

Quantification of positive granulosa cell

Three sections were used to quantify positively stained granulosa cells, all follicles were counted for positive stained target antibody with Olyvia (Olympus) software, and only the positively stained GCs relative to total GCs cells were statistically analyzed.

[1] Habara O, et al. WNT signaling in pre-granulosa cells is required for ovarian folliculogenesis and female fertility. Development (Cambridge, England), 2021, 148(9)
